# The Emerging Proteomic Research Facilitates in-Depth Understanding of the Biology of Honeybees

**DOI:** 10.3390/ijms20174252

**Published:** 2019-08-30

**Authors:** Solomon Zewdu Altaye, Lifeng Meng, Yao Lu, Jianke Li

**Affiliations:** 1 Institute of Apicultural Research/Key Laboratory of Pollinating Insect Biology, Ministry of Agriculture, Chinese Academy of Agricultural Sciences, Beijing 100081, China; 2 Agricultural Information Institute, Chinese Academy of Agricultural Sciences, Beijing 100081, China

**Keywords:** honeybee proteomic research, molecular basis, research themes and trends, honeybee biology

## Abstract

Advances in instrumentation and computational analysis in proteomics have opened new doors for honeybee biological research at the molecular and biochemical levels. Proteomics has greatly expanded the understanding of honeybee biology since its introduction in 2005, through which key signaling pathways and proteins that drive honeybee development and behavioral physiology have been identified. This is critical for downstream mechanistic investigation by knocking a gene down/out or overexpressing it and being able to attribute a specific phenotype/biochemical change to that gene. Here, we review how emerging proteome research has contributed to the new understanding of honeybee biology. A systematic and comprehensive analysis of global scientific progress in honeybee proteome research is essential for a better understanding of research topics and trends, and is potentially useful for future research directions.

## 1. Introduction

Honeybees play an essential role in plant pollination and provide valuable service to the ecosystem [1]. One-third of the world food production relies on insect pollinators, mainly honeybees [2]. Managed colonies of some of the honeybee species such as *Apis mellifera* are kept for their products such as honey, royal jelly, and beeswax. The social and instinctive behavioral characteristics of honeybees, together with their body structure, makes them efficient pollinators [3], which also contributed to their evolutionary success [4]. Despite the crucial role of honeybees in the agricultural economy and ecosystem services, it was only at the beginning of the 20th century that studies investigating honeybee biology at the molecular and biochemical levels started.

Unlike other insects like *Drosophila*, honeybees were poorly studied at the molecular, biochemical, and cellular level, which is mainly associated with the knowledge and technical limitations. On the other hand, for a long time, honeybees have been used as model organisms for the research and understanding of social behavior. However, studies are merely explored at the molecular level in developmental biology, neurobiology, immunology, and aging [5]. It is the advent of the completion of honeybee genome sequencing in 2006 [6] that opened doors for the functional genomic studies of the honeybee. In the process of understanding honeybee biology, the honeybee genome sequence is fundamental but not sufficient to reveal all of its intricacies. Hence, the study of gene expression through proteomics becomes necessary, as genome sequence and protein functions are not directly related.

Proteomic research on honeybees has substantially expanded due to rapid technological developments in the last decade in term of mass spectrometry (MS), protein separation, and bioinformatics algorithm. The earliest honeybee proteomics study that was published in 2005 identified 9 proteins from the venom of honeybees using a two-dimensional gel electrophoresis (2-DE)-based method [7]. A year later, when the first bee genome was released, another bee proteomic study that was conducted using one-dimensional gel electrophoresis (1-DE) plus MS-based proteomics identified 324 proteins in the hemolymph of queens, drones, workers, and worker larvae, which found a profound proteome difference among the castes [8]. Most of the earlier studies on honeybee proteome analysis mainly depended on the 2-DE-based proteomics, however, due to the fairly insensitive and low reproducible nature of the 2-DE, only a small fraction of proteins in single honeybee samples were identified [9,10,11]. Furthermore, working on the abundant data generated using 2-DE-based proteomics is tedious, and above all the quality of the results obtained from the identification and quantification of spectra is subject to semi-manual analysis, which imposed challenges on the early proteomic studies of the honeybee [12]. Through time, the field of proteomics has moved towards more powerful liquid chromatography coupled tandem MS (LC-MS/MS) methods that have dramatically improved in speed and quality, such as in resolution, sensitivity, and mass accuracy, enabling large scale analysis of proteins [13,14]. For instance, protein expression atlases for the adult stage of all three castes of *Apis mellifera* from 29 different organ/tissue types have been analyzed, and 2288 proteins have been identified [15]. Recently, >8600 proteins were also identified across samples of the hemolymph, mushroom body, and antenna of honeybees using the LC-MS/MS method, which has considerably improved the depth of the protein coverage [16]. At the current stage, there is a wide range of insight into the molecular basis of social behaviour and physiology of honeybees [5,17]

After more than a decade of progress in bee proteomics research, a comprehensive analysis of global scientific production is essential for a better understanding of research topics and trends. Here, we summarized the proteomic research in the new understanding of honeybee biology. New depths at the molecular and biochemical level have been attained for a wide range of honeybee biology such as developmental biology, physiology, behavior, neurobiology, and immunology by using the proteomic approach. The information from these studies helps to determine the breadth of bee proteome research and to define further research directions.

## 2. Proteomics Unravels the Molecular Basis for a Wide Range of Honeybee Biology

Proteomics is becoming a powerful tool to reveal the molecular basis of honeybee biology [5]. The different organs and tissues that are required for honeybees to perform their biological tasks have been explained at proteomic scale, for instance, in the brain [15,18,19,20,21], hypopharyngeal gland (HG) [22,23], mandibular gland (MG) [24], hemolymph [25,26], embryo [27,28], venom [29,30,31], antennae [16,32], and long-term storage adaptation of honeybee sperm [33,34,35,36,37]. In addition, in recent years, extensive research has been conducted to reveal the molecular underpinnings for the enhanced performance of HG in secretion of royal jelly (RJ) by a honeybee stock: royal jelly bees (RJBs), that has been selected for increasing royal jelly production [17,23,38]. Detailed descriptions of how emergent proteomics research contributed to the new understanding of honeybee biology are given in the following sections.

### 2.1. The Molecular Basis Behind a Complex Social Immunity Behavior

Different bee species develop significant variations in the hemolymph proteome to support their respective physiology. In 2006, Chan and his colleagues presented the first molecular visualization of the honeybee hemolymph [8]. They focused on the differences in the hemolymph composition between bee castes, and found differences between the larvae and adult stages, between male and female castes, and between adult workers and the queen bees. Especially compared to larvae, adult workers have more immune-related proteins in their hemolymph [8]. Proteome of larval hemolymph across ages is compared to investigate the susceptibility of bees to major age-related infectious bee diseases such as American Foulbrood or chalkbrood [10,39]. Between sterile and reproductive workers, the hemolymph proteome reveals differences in viral protein load in their hemolymph [40,41], and the reproductive workers develop a stronger immune system than the sterile ones [40]. Furthermore, comparison between the hemolymph proteome of adult workers and larvae has found a high abundance of vitellogenin in the hemolymph of adult workers, which provides a vital contribution to disease resistance [42].

Proteomics has also gained new molecular insight into complex social immunity behaviors: the reason why bees co-exist with pathogens. For instance, comparative hemolymph proteome between *A. m. lingustica* and *A. c. cerana* has revealed that both bees develop unique hemolymph proteome architecture for nutrient transport and immune defence during larval to pupal development [25]. The western and eastern bees have evolved unique olfactory mechanisms during the course of evolution to fit a different environmental condition, which are ecological and pest-resistant behavioral adaptations [43]. In addition, the *Varroa*-sensitive hygienic (VSH)-line, a bee-line selected from *A. m. carnica* for its VSH behavior relative to non-VSH bees [44,45], enhances its level of energy metabolism and protein synthesis during pupal organogenesis to cement social immunity [16]. Moreover, the antennae of VSH bees associated with olfactory senses and signal transmissions are functionally enhanced to boost the roles of transmitting signals to mushroom bodies to initiate VSH behavior [16]. Again, proteome analysis on disease-tolerance behavior, colony-level hygienic behavior and VSH behavior have identified highly predictive proteins that contribute to reducing hive infections by changing the antennal proteome of hygienic bees [32]. These changes in the hygienic bee antennal proteome provide specificity for detecting the *Varroa destructor* in the antenna, thereby stimulating the speed of hygienic behavior by promoting chemosensory and neural processes [32]. All these findings have significantly expanded our understanding of the molecular underpinnings behind complex social immune behaviors which allow bees to coexist with pathogens. Therefore, the genetic and biochemical factors that drive these adaptations need to be further investigated.

### 2.2. Understanding of Biochemistry and Function of Honeybee Venom

Honeybee venom proteomics study gains novel understanding of its biochemical components and functions. Honeybee venom is a complex toxin composed of a variety of biological and pharmacologically active substances, such as biogenic amines, peptides, and proteins [31,46]. Honeybee venom is used as a chemical weapon to protect the colonies and food, mainly from vertebrate predators [31]. Proteomic studies on honeybee venom explores active proteins as allergens and toxins [30]. Venom research may also provide potential targets for new drugs and provide a better understanding of molecular structure and function [47]. The first proteomic work on honeybee venom protein composition revealed three protein domains [7]. Comparative proteome and phosphoproteome analysis of honeybee venom collected from electrical stimulation and manual extraction reveal three new phosphorylated venom proteins in electrical stimulation that may cause different immune responses by specific identification of the determinants of the antigenic [48]. Similarly, the proteome and phosphoproteome of Africanized honeybee venom are compared with two European subspecies, namely *A. m. ligustica* and *A. m. carnica* venom, and 51 proteins are identified, and phosphorylation of two toxins, melittin and icarapin, is carried out [49]. In all the venom, the icarapin is phosphorylated at the 205 Ser residue, and melittin, the main toxin of bee venom, is phosphorylated at the 10Thr and 18Ser residues. The 18Ser phosphorylated melittin is the main form of its two phosphorylation forms and is less toxic than the native peptide [49]. In addition, the exploration of the hidden honeybee venom proteome identified 102 venom proteins and peptides, 33 of which are putative venom toxins [50]. This greatly expands our understanding of venom toxicity [50]. Moreover, the proteome of queens and winter bees venom reveals 34 venom toxins, of which 2 are novel [51]. The study points out that, although barely any people are stung by winter bees or queen bees, these newly discovered toxins should be taken into account when describing the allergic reaction to *Apis mellifera* stings [51].

The venom proteome of *Apis mellifera* emphasizes the complementarity of analytical methods in improving the detection ability of honeybee venom proteins [30,31]. A total of 269 types of proteins, 49 of which are bee toxins, allergens and their constituents, which are involved in the toxin mechanism of envenoming and belong to toxins, proteases/peptidases, protease inhibitors, hydrolases, and major royal jelly proteins (MRJPs) [30]. In addition, five other putative toxins have been identified [30]. In another study, the proteomic analysis of the venom of *Apis mellifera* identifies 394 peptides, which provides the identification of 50 components, including putative toxins and trace elements, the recognition of 12 known allergens of bee venom, and the detection of 4 new hypothetical proteins [31]. All of the peptides identified in this study belong to the esterase group and are putative toxins and allergens. For instance, phospholipase A2 is the major allergen of bee venom, with up to 97% of patients allergic to venom having specific antibodies against Api m1 [31]. It can be concluded that advances in instrumentation and analytical techniques provided a deeper exploration of bee venom composition. Here, identification of the functions of some of the uncharacterized compounds can contribute to exploration of possible new venom allergens [31]. Thus, further research is needed to determine how many of the proteins/peptides have a venomous effect to which organism and what kind of effect.

### 2.3. The Molecular Basis of Neurology Implicated in the Behavior of Honeybees

The age-related behavioral transition in honeybee workers is closely linked to changes in the structure, gene expression, and protein synthesis of the brain [52]. Age-dependent polyethism (task specialization of honeybee workers) is regulated in a sophisticated and diverse manner, associated with social influences, pheromones, environmental disorders, and endocrine regulation of vitellogenin titers and nutritional status associated with their physiological status [53,54,55,56]. Gene expression changes in the honeybee brain can be used as an indicator of naturally occurring behavior [57,58]. At the same time, the honeybee brain proteome is closely related to the age-related transition from working in the hive to foraging [59,60]. In addition, the dynamics of neuropeptides in the brain of honeybees are related to the regulation of social behavior [18].

Understanding the molecular neurobiology is the basis for differences in behavior between and within honeybee species. The proteomes of three brain regions (mushroom bodies, optical lobes, and antennal lobes) of *A. m. ligustica* and *A. c. cerana* have revealed that they evolve similar proteome characteristics in both mushroom body and optical lobe to drive domain-specific neurological activity. However, they develop unique antennal lobe proteome structures to provide the basis for their respective olfactory learning and memory [20]. On the other hand, compared to Italian bees (ITBs) or *A. m. lingustica*, high RJ-yielding strain of bees (RJBs) that are selected from ITBs have enhanced the neuropeptide to regulate behaviors such as brood pheromone recognition, water homeostasis, foraging efficiency, which is vital for an enhanced level of RJ secretion [18]. Similarly, phosphatidylinositol signalling and arachidonic acid metabolism are essential in a stronger olfactory sensation for the RJB nurses in response to larval pheromone stimulation, and pathways related to signal processing are to improve neurological sensitivity in RJB foragers to promote efficient pollen collection [18,19]. Furthermore, in connection with VSH behavior, the VSH bee’s neurological sensitivity to non-VSH bees involves a higher level of mushroom body proteins expression, which may support the performance of VSH behavior by initiating synaptic vesicles and calcium channel activity [16].

The honeybee brain reveals the molecular basis associated with neural function related to foraging. For instance, the first 2-DE-based proteomics finds the juvenile hormone diol kinase (JHDK) is abundant in the mushroom bodies of worker bees, while glyceraldehyde-3-phosphate dehydrogenase (GAPDH) is selectively expressed in optical lobes. This suggests that the role of JHDK in the formation of juvenile hormone diol phosphate is to promote Ca^2+^ signalling in the mushroom bodies and GAPDH is to promote glucose metabolism [61,62]. Moreover, the levels of proteins associated with kinases, synapses, and nerve growth in the central brain decrease with increasing foraging duration, while the mushroom body calyx region remains unchanged in structure and biochemistry [63]. As a result, different brain regions are affected differently by the biological aging of bees, and it is proposed that the calyx region is not responsible for the decline in foraging dependence performance [63]. While the brain function of the foraging bees decreases with age, the ability to learn can be restored among aged foraging bees that revert to a nursing task, which is directly related to the stress response/cell maintenance proteins level in the central brain [64].

The molecular mechanisms of olfactory learning and memory underpins the biochemical and physiological changes in honeybees. For instance, the proteomic complement is associated with the proboscis extension reflex to promote understanding of the major molecular transformations in the honeybee brain during the execution of an unconditioned stimulus [65]. In the brain of proboscis extension reflex-stimulated individuals, the metabolism of cyclic/heterocyclic/aromatic compounds is simultaneously activated with the metabolism of nitrogen-containing compounds, followed by up-regulation of carbohydrate metabolism, proteins involved in anatomy and cytoskeleton; down-regulation of anatomical development and cell differentiation in neurons [65]. By reproducing this strategy, the learning process and memory acquisition of the bees can be better understood. In another study, the effects of fipronil (a phenylpyrazole insecticide) on the brains of Africanized *Apis mellifera* workers are investigated, and the toxico-proteomic analysis of newly emerged and aged bee workers’ brains exposed to sublethal doses (10 pg fipronil per day for 5 days), and non-exposed groups are compared [66]. Proteomic analysis identified 25 proteins that are up- or down-regulated when comparing the fipronil exposed and non-exposed groups. These proteins may be associated with pathogen susceptibility, chemical stress in neurons, misfolding of neuronal proteins, and apoptosis, ischemia, visual impairment, impaired synapse formation, brain degeneration, memory, and learning disabilities. Exposure of bees to very low doses of fipronil has been short enough (5 days) to elicit many important neuroproteomic changes in honeybee brains [66].

### 2.4. The Molecular Biology Behind the Honeybee Pre-Adult Developments

The molecular biology behind the honeybee pre-adult developments has been extensively investigated using proteomics. For instance, an early proteomic study in the honeybee larvae found 22 upregulated proteins that are mainly involved in carbohydrate metabolism, suggesting carbohydrate metabolism is needed for the young larval development [10]. On day 6, higher levels of storage proteins such as larval serum protein 2 indicate that larvae store amino acids for subsequent metamorphosis [10,39]. During the embryonic development of *A. mellifera* worker bees, 38 proteins that are involved in cell growth and differentiation are identified using 2-DE-based proteomics [67]. Embryogenesis in honeybees is a first priority process, and most of the proteins accumulated at this stage are vital for cell division, tissue metamorphosis, and self-protection [28]. Compared to 2-DE-based proteomics in embryogenesis, the MS-based works have identified 1460 proteins across three ages (24, 48 and 72 h) [28]. The proteome at each time point matches the development events of embryogenesis [28]. Proteome analysis of the physiological transition from egg to larvae has shown the same trend [68,69].

Compared to drones, worker embryos begin the morphogenesis later, and the protein abundance associated with morphogenesis in workers’ embryos is lower throughout the embryonic process [27]. In addition, drone embryos use more cytoskeleton proteins to support their large body size and use antioxidants to support their temporal organogenesis [27]. The proteome variations between freshly collected bee embryonic cells and cultured ones indicate that most of the proteins are at a higher level in cultured cells in order to adapt to unnatural environments [70]. As early as the third larval instar, proteins support the time point of nutritional switch to the fate of caste. For example, significant variances in protein expression between the queen intended larvae and worker-intended larvae in 72 hours and 120 hours suggest that the fate of the two castes is determined before 72 hours [71]. In addition, the subcellular proteomics (mitochondria and nuclei) of larvae indicate that there are significant variations in protein expression between the two castes intended larvae at the age of day 3, day 4, and day 5, showing a strong directivity of selective pressure due to the quantity and quality of larval foods provided throughout the developmental phase [72,73]. Interestingly, this sub-cell proteome evidence is consistent with the findings of proteomics analysis on the whole larvae [71]. In addition, royalactin can induce bee larvae to differentiate into queens and derive queen development through signalling pathways mediated by epidermal growth factor receptors [74].

In honeybees, the non-feeding pupal stage is the longest post-embryo development period, lasting ~13 days [75]. A series of changes occur in the body structure and pigmentation of the compound eye during this period [75]. The earliest proteomic work on pupal heads using 2-DE found 58 differentially expressed proteins at five points in time (13, 15, 17, 19 and 20 days), of which 36 proteins were involved in the organogenesis of the head occurring in early development [76]. However, 22 of these proteins were involved in regulating the development of pupal head neurons and glands that occurred later in the development of the pupae head [76]. Furthermore, of the identified ∼129 proteins by 2-DE proteomics of worker red-eye pupae hemolymph [77], most of the proteins during non-feeding stage are related to physiological changes during the metamorphosis [77,78], and as red-eyes develop into a newly emerged bee, protein quality drops significantly [78].

### 2.5. Social Ontogeny of Adult Workers

Whole body proteome change of worker bees is likely to fit the biological function of nurse bees and forager bees [79]. Significant differences between foraging bees before and after reversal from foraging to nursing activity suggest that the plasticity and robustness of upregulated protein expression are related to changes in the physiological and behavioral roles of worker bees [80]. The metabolic specialization that occurs during the individual social development of worker bees is to meet their metabolic energy needs and utilization, as worker bees begin to fly intensively during foraging [79,81]. Compared to the brains of nurse bees, the over-expressed proteins in experienced foragers are involved in energy production, iron binding, metabolic signalling, and neurotransmitter metabolism, which further supports the social ontogeny of worker bees [59]. Similar proteome analysis of the brains of foragers and nurse bees also demonstrates that proteins involved in energy production and transformation are elevated in experienced foragers, suggesting that increased brain activity needs to be acquired during learning and memory [60]. However, as opposed to foragers, nurse bees enhance activity involved in translation, ribosome structure, and biogenesis, thereby developing the protein machinery necessary for structural changes in the brain [60]. Furthermore, in addition to the role in regulating caste differentiation, the differential expression patterns of the MRJPs (MRJP1, MRJP2, and MRP7) in the brains of nurses indicate that they may be endogenous participants in various brain activities [82].

### 2.6. The Molecular Basis for Enhanced RJ Production in RJB

A deeper molecular insight into the enhanced performance of RJ secretion by RJBs has been gained [17,38]. China has been working on the selection of a high RJ-yielding strain of bees -RJBs from ITBs. RJB is the world’s most important RJ producer, with production accounting for more than 90% of the total production and an annual market value exceeding $2.5 billion. With the advancement of proteomics technology, the molecular basis of RJB high RJ production has been explored to new depths in the past decade.

Compared to ITB, the HG of RJBs has evolved a wider range of pathways that can increase RJ production by 10-fold [22]. For example, in the HG of RJB, pathways such as carbohydrate metabolism, protein biosynthesis, and protein folding are induced to increase glandular activity, thereby increasing the secretion of RJ [22]. Similarly, compared to ITB, in the HG of royal jelly nurse bees, pathways for energy metabolism and protein synthesis are functionally induced to consolidate augmented RJ secretion [23]. The phosphoproteome analysis of HG at each age of worker bees also showed that phosphorylation could contribute to increasing the HG activity of worker bees [83]. The MGs of the worker bee is used for the secretion of lipids for larval nutrition and pheromones. Proteome analysis of worker MG of RJB and ITB across age groups (newly emerged bees, nurse bees, and forager bees) reveals a wide range of biological processes that define different programs to underline the specific role of the sub-castes in the colony [24]. For instance, in RJB and ITB emerging nurses and foragers, the MGs use different proteome settings to accommodate their unique age-dependent physiology such as the rate of lipid synthesis and the reduction of its degradation to increase 10-hydroxy-2-decenoic acid (10-HDA) synthesis [24]. The MG of RJB also induced pathways associated with lipid synthesis to maintain an appropriate ratio of 10-HDA, an important fatty acid in RJ for feeding larvae, and also contributing to the amount of RJ produced [24].

Hemolymph plays a role in studying various aspects of the physiology and phenotype of honeybees [8,25,78]. For instance, hemolymph proteins such as enzymes, immune response proteins, structural proteins, nutrients and pheromone transporters, and MRJP differ between castes, and stages of development, physiological and developmental stages [8,84]. To better understand the molecular basis of Royal Jelly bees enhanced royal jelly production, a comparison of the hemolymph proteome between RJB and ITB larvae and adult samples has been made and indicates that the two bee stocks use different hemolymph proteomes in driving their physiology [26]. In particular, on day 4, the RJB larvae hemolymph proteome consolidates amino acid and protein synthesis to support developmental and immune responses, which drives selection for high RJ production, RJB nurse bees enhance energy metabolism, protein synthesis, and homeostasis in response to increased RJ production [26].

Brain membrane proteome and phosphoproteome comparison of RJBs and other honeybee species/lines reveal the neurobiological basis of elevated RJ production. Both the membrane proteome and the phosphoproteome have evolved a unique setting to accommodate the increase in RJ secretion of RJB [18,19]. RJ is a secretory mixture of HG, MG, and thorax, and the former two glands reshape their proteome to promote the development and function of the glands, thereby enhancing the production of RJ. Similarly, the RJB nurse’s brain has evolved a unique neuropeptide to support the increased RJ production, as RJ secretion is a behavior performed by nurse bees. For instance, in RJB, enhanced level of neuropeptides associated with regulating water homeostasis, brood pheromone recognition, foraging capacity, and pollen collection indicate their roles in the regulation of RJ secretion [18]. Similarly, at each stage of ITB and RJB, identified membrane proteins are enriched in similar pathways [19]. The upregulated proteins and phosphoproteins in the brain also explain the enhanced mechanism by which RJB secretes RJ well [18,19]. In addition, immune staining of the brain and HG showed different MRJP expression in different brain regions of honeybee castes and subcastes [85], suggesting that the level of activity in the brain may have led to an increase in RJB’s RJ yield compared to ITB. In conclusion, in RJBs, the trait of high RJ production is associated with several important proteins and pathways. However, the specific activity of these proteins in related pathways remains to be further studied.

### 2.7. Proteome, Phosphoproteome, and Glycoproteome Analyses of RJ from Different Bee Species or Strains Reveal the Biological Significance to Honeybees

Royal jelly is a cocktail of three different glandular secretions (i.e., HG, MG, and thorax) of worker bees at the age of 5 to 15 days [86]. Most proteins of the MRJP family are derived from HG [87], while MG produces fatty acids [88]. In addition to their expression in HG, MRJPs have been found in different parts of the body, including the head, thorax, and abdomen of nurse bees, forager bees, and drones [89,90]. Proteins are the most important component *of which* MRJP family are the biggest/most important [91]. Nine major royal jelly proteins (MRJP1-9) have been described, with MRJP-1 being most abundant in RJ [91,92]. MRJPs accounted for 82–90% (w/w) of the total protein in RJ [92,93,94]. Moreover, proteins such as 3-glucose oxidase, 1-peroxiredoxin, and 1-glutathione-S-transferase were identified in three RJ samples of ITBs, RJBs, and Carnica bees using 2-DE [95]. It was also found that the RJ protein compositions were the same among the three species, but the number of proteins detected in Carnica bees was significantly lower [95]. The biological and pharmaceutical properties of RJ are mainly attributed to protein and its peptides, fatty acids, phenolic compounds, and free amino acids [96,97].

The RJ proteome and function from the two bees *A. m. ligustica* and *A. c. cerana* showed great differences. Although the protein types were the same, MRJP levels were significantly higher in *A. m. ligustica*-RJ than in *A. c. cerana*-RJ [98], phosphorylated protein abundance levels showed a reversed trend, while phosphorylated peptides from *A. c. cerana*’s RJ showed stronger anti-microbial and anti-fungal activity [99]. The abundance levels of MRJP in both species are better explained by their biological requirements for survival and development. For example, in *A. m. ligustica*, the frequency of MRJPs is associated with the support of their large body size, while in *A. c. cerana* a high frequency of phosphorylated peptides compensates for the low MRJP levels to sustain survival and development [99]. In addition, RJ protein complements showed significant variation between Carnica bees and ITBs or RJBs, but between the latter two, no significant variations were observed [95]. However, questionable results were found on the RJ-10-HDA content. One study showed that RJ from RJB colonies had a relatively low 10-HDA content compared to RJ from ITB colonies [100], while in another study 10-HDA was reported to have a non-significant content difference between two lines [24].

Comparative proteome, phosphoproteome, and glycoproteome studies reveal various biological properties of RJ from RJB and other honeybee species. Using two-dimensional gel electrophoresis-based proteomics, a total of 52 and 60 proteins are identified in the RJ of *A. m. ligustica* and *A. c. cerana*, respectively [98]. Significantly abundant protein species are found in the RJ of RJBs relative to *A. c. cerana* [98]. For instance, proteins such as MRJP5, peroxidase 2440, and glutathione S-transferase S1 are only identified in *A. m. ligustica*-RJ, and MRJP1 is the most abundant MRJP. MRJP7 was the only protein found in *A. c. cerana* -RJ, and similar to *A. m. ligustica*-RJ, MRJP1 is the most abundant protein. In addition, the level of MRJP1-5 protein in *A. m. ligustica*-RJ was significantly higher than that of *A. c. cerana*-RJ [98]. Moreover, in 2011, a total of 37 and 22 non-redundant *A. m. ligustica*-RJ proteins are identified using gel-based and shot-gun proteomic approaches, respectively, and an additional 19 new proteins were identified [101]. These newly identified proteins are mainly classified into three functional categories: oxidation-reduction, protein binding, and lipid transport. In 2014, Zhang and his colleague from our research group identified 13 novel proteins that have activities mainly related to metabolic processes and heal improvement [102]. In addition to extending the coverage of the RJ proteome, newly identified proteins provide new information about the biochemical properties of RJ, as the identification and characterization of unknown RJ proteins may be useful for pharmacokinetics and biological activity [102], and future research should proceed to identify potential proteins for such use.

The different biological properties of *A. c. cerana* and RJB are supported by the unique phosphorylation strategy they pursue [99]. Both species showed significant variation in the abundance of peptides, phosphosites, and the antimicrobial activity of phosphorylated RJ proteins. In *A. m. ligustica*-RJ, 16 phosphoproteins with 67 phosphorylation sites were identified, while in *A. c. cerana*-RJ 9 phosphorylated proteins were found at 71 sites. Of the two RJ samples, eight phosphorylated proteins were common and the same motif was extracted ([SxE]), indicating that in both bee species the function of MRJP as a nutrient and immunological substance was evolutionarily preserved [28]. However, the frequency of these eight overlapping phosphoproteins is higher in *A. c. cerana*-RJ than in *A. m. ligustica*-RJ. In addition, in *A. c. cerana*-RJ, the phosphorylation of Jelleine-II (an antibacterial peptide TPFKLSLHL) at S6 shows stronger antimicrobial properties than at T1 in *A. m. ligustica*-RJ [28].

In RJ, glycosylation regulates various biological processes that are important to both bees and humans. Several N-glycosylated proteins found in RJ are associated with MRJPs, developmental regulation, metabolic processes, and immunity activities [103]. For instance, in *A. m. ligustica*-RJ, 80 nonredundant *N*-glycoproteins harbors 190 glycosites, of which 23 novel proteins carrying 35 glycosites; for *A. c. cerana*, 43 proteins are N-glycosylated at 138 glycosites [103]. Due to species-specific glycosylation of RJ, the two bee species have similar but different functional properties, for instance, in the inhibition efficiency of honeybees against *Paenibacillus larvae* (*P. larvae*) and in the treatment of hypertensive diseases [103]. Specifically when compared to *A. c. cerana*-RJ, the *A. m. ligustica*-RJ larvae are susceptible to *P. larvae* due to the lack of antibacterial hymenoptaecin, glycosylated apidaecin, and peritrophic matrix, and the inhibition efficiency of N-glycosylated MRJP2 is low against *P. larvae* [103]. In addition, greater antihypertensive activity of N-glycosylated MRJP1 was found in *A. c. cerana* than in *A. m. ligustica*, demonstrating the purpose of the specific RJ protein and modification for the treatment of hypertensive disease in humans [97,103]. In general, the two honeybees have developed species-specific strategies of glycosylation to tailor protein activity as a nutrient and immune substance, benefiting both the honeybee and enhancing human health-promoting activity. This evidence is a valuable resource for studying the biological functions of RJ proteins in the honeybee and for medical industries.

### 2.8. Antennal Proteomics Reveal the Olfactory and Neural Activity of Honeybees

As a social insect, bees have a comple×smell cue (pheromone) communication system that relies on odor signals to attract their potential partners, find food sources, and discover possible enemies [104]. In bees, the reception of odor signals carries out through the placode sensilla in the antenna and binds to the odorant binding protein (OBP) and the chemical sensory protein (CSP) to activate the subsequent neural response and eventually achieve its social activities [105]. The first antenna proteome study focused on individual expressions of OBP and CSP, has revealed the presence of four OBP (OBP1, OBP2, OBP4, and OBP5) and two CSP (CSP1 and CSP3) in the forager bee antennae of *A. mellifera* [105]. On the other hand, a comprehensive antennal proteome comparison between the forager and the drone bee has found a gender-specific odor response mechanism because of natural selection for different olfaction in the two castes [106]. A more comprehensive proteome comparison between drones, worker bees, and queen bees confirms again that the differential expression of the antennal protein is associated with different requirements depending on the olfactory activity of the castes [107]. Recently, the correlation between the expression pattern of individual proteins and the hygienic behavior score found that 7 proteins may be putative biomarkers of hygienic behavior [108]. All of these proteins are also involved in the transmission of neuronal signals semichemical perception and signal decay. Similarly, the upregulated proteins in the antennae of VSH bees suggest that antenna proteins play an essential role in transmitting important signals to the mushroom body to activate VSH behavior in VSH bees [16]. Thus, these findings provide molecular clues to the social immune responses that allow bees to coexist with pathogens and may contribute to future protein-based selective breeding to better resist Varroa.

### 2.9. Molecular Basis for Long-Term Storage Adaptation of Honeybee Sperm

The biochemical compositions and physiological adaptations that contribute to the extended survival of sperm has been revealed [33,34,35,36,37]. The first proteomic work on semen proteins was conducted in 2006 by Collins and his colleagues, and they found biases in metabolism-associated proteins in semen vs. the seminal vesicle, which suggests the specific metabolic and catabolic activities that sustain sperm prior to and during mating [109]. In another study, comparative proteome analysis between male accessory gland-derived seminal fluid and the spermathecal gland secretions indicate male accessory gland-derived seminal proteins enhances survival of sperm, while the secretory proteins of spermathecal glands have a comparable positive effect on the viability of sperm [33]. Moreover, proteins in the spermathecal fluid are mainly enzymes of antioxidant defense and energy metabolism, showing that this fluid may promote sperm storage for a long period of time [35]. Intraspecies of *A. mellifera* seminal fluid contains a similar set of proteins, but the protein abundance or condition of protein modifications vary significantly [34].

The seminal fluid of honeybees contains enzymes, regulators, and structural proteins used for energy production, and antioxidants that interact with female physiology and maintain sperm stability and viability [33,34,37,110]. Proteins with altered abundances have different biological functions and are associated with male reproductive success, energy metabolism and cellular structure proteins, and immunity [34]. The long-term survival of sperm can be supported by significant changes in only a specific subset of sperm proteins, thus allowing physiological adaptation to storage [36]. However, due to the plasticity of sperm cell mechanics between ejaculation and sperm in female storage organs [110], there is not enough evidence whether changes in protein abundance are due to active adaptation or sperm senescence. It also remains unclear whether these proteins can adapt the performance of sperm to different chemical environments, and it is necessary to be further investigated.

## 3. Future Research Directions

The global academic community is rapidly conducting honeybee proteome research and significant progress has been made in uncovering the molecular basis of honeybee biology. For example, several expression-based proteomic changes have been discovered that reach new depths at the molecular and biochemical levels in broad areas of honeybee biology, such as developmental biology, physiology, behavior, neurobiology, and immunology. Future research should focus on the diversity of molecular mechanisms and social functions to broaden our understanding of the cellular and molecular levels of honeybee development and behavioral physiology. A novel understanding of the molecular basis of honeybee development and behavioral physiology helps us identify critical proteins/genes and signaling pathways for the downstream mechanistic investigation by knocking a gene down/out or overexpressing a gene and attributing it to a specific phenotype/biochemical change.
